# Bayesian methods: a potential path forward for sepsis trials

**DOI:** 10.1186/s13054-023-04717-x

**Published:** 2023-11-08

**Authors:** George Tomlinson, Ali Al-Khafaji, Steven A. Conrad, Faith N. F. Factora, Debra M. Foster, Claude Galphin, Kyle J. Gunnerson, Sobia Khan, Roopa Kohli-Seth, Paul McCarthy, Nikhil K. Meena, Ronald G. Pearl, Jean-Sebastien Rachoin, Ronald Rains, Michael Seneff, Mark Tidswell, Paul M. Walker, John A. Kellum

**Affiliations:** 1 Dalla Lana School of Public Health, University of Toronto, Toronto, ON Canada; 2 Department of Critical Care Medicine, University of Pittsburgh, 3550 Terrace Street, 600 Scaife Hall, Pittsburgh, PA 15261 USA; 3 Departments of Medicine, Emergency Medicine, Pediatrics and Surgery, Louisiana State University Health, Shreveport, LA USA; 4 Department of Intensive Care and Resuscitation, Cleveland Clinic, Cleveland, OH USA; 5 Spectral Medical Inc, Toronto, ON Canada; 6 Southeast Renal Research Institute, CHI Memorial Hospital, Chattanooga, TN USA; 7 Departments of Emergency Medicine, Anesthesiology, and Internal Medicine, University of Michigan Medical Center, Ann Arbor, MI USA; 8 Department of Medicine, Stony Brook University Hospital, Stony Brook, NY USA; 9 Institute for Critical Care Medicine, Icahn School of Medicine at Mount Sinai, New York, NY USA; 10 West Virginia University, Heart & Vascular Institute, Morgantown, WV USA; 11 Division of Pulmonary and Critical Care Medicine, University of Arkansas for Medical Sciences, Little Rock, AR USA; 12 Department of Anesthesiology, Perioperative and Pain Medicine, Stanford University, Stanford, CA USA; 13 Cooper University Healthcare, Cooper Medical School of Rowan University, Camden, NJ USA; 14 Pulmonary Associates, Univ of Colorado Health-Memorial Hospital, Colorado Springs, CO USA; 15 Department of Anesthesia and Critical Care, George Washington University Hospital, Washington, DC USA; 16 Pulmonary and Critical Care Division, Baystate Medical Center, Springfield, MA USA

**Keywords:** Septic shock, Endotoxemia, Endotoxin septic shock, Statistical methods, Polymyxin-B, Hemadsorption, Trial simulation

## Abstract

**Background:**

Given the success of recent platform trials for COVID-19, Bayesian statistical methods have become an option for complex, heterogenous syndromes like sepsis. However, study design will require careful consideration of how statistical power varies using Bayesian methods across different choices for how historical data are incorporated through a prior distribution and how the analysis is ultimately conducted. Our objective with the current analysis is to assess how different uses of historical data through a prior distribution, and type of analysis influence results of a proposed trial that will be analyzed using Bayesian statistical methods.

**Methods:**

We conducted a simulation study incorporating historical data from a published multicenter, randomized clinical trial in the US and Canada of polymyxin B hemadsorption for treatment of endotoxemic septic shock. Historical data come from a 179-patient subgroup of the previous trial of adult critically ill patients with septic shock, multiple organ failure and an endotoxin activity of 0.60–0.89. The trial intervention consisted of two polymyxin B hemoadsorption treatments (2 h each) completed within 24 h of enrollment.

**Results:**

In our simulations for a new trial of 150 patients, a range of hypothetical results were observed. Across a range of baseline risks and treatment effects and four ways of including historical data, we demonstrate an increase in power with the use of clinically defensible incorporation of historical data. In one possible trial result, for example, with an observed reduction in risk of mortality from 44 to 37%, the probability of benefit is 96% with a fixed weight of 75% on prior data and 90% with a commensurate (adaptive-weighting) prior; the same data give an 80% probability of benefit if historical data are ignored.

**Conclusions:**

Using Bayesian methods and a biologically justifiable use of historical data in a prior distribution yields a study design with higher power than a conventional design that ignores relevant historical data. Bayesian methods may be a viable option for trials in critical care medicine where beneficial treatments have been elusive.

**Graphical abstract:**

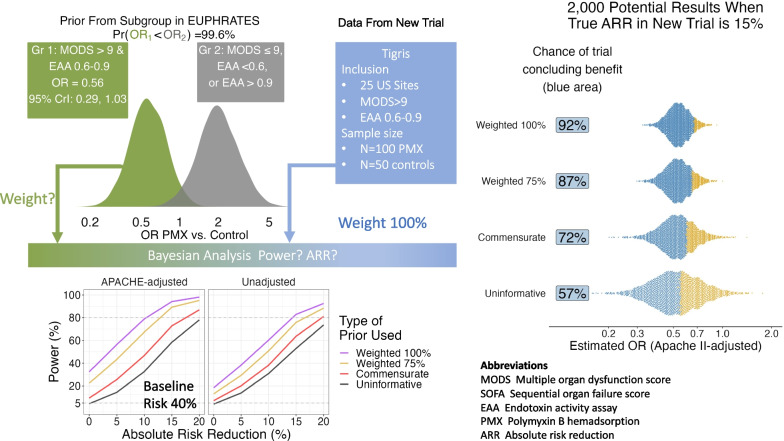

**Supplementary Information:**

The online version contains supplementary material available at 10.1186/s13054-023-04717-x.

## Background

Sepsis is defined as life-threatening organ dysfunction caused by a dysregulated host response to infection [1]. While effective interventions for infection are available, treatments for sepsis have been elusive perhaps because no single underlying biologic process can account for the range of severity and distribution of organ failures encountered in sepsis. This variation is directly associated with hospital mortality which ranges from 2 to 32% [2].

Heterogeneity of the phenotype that defines sepsis is a significant problem for clinical trials and the problem cannot be solved by increasing the total sample size—the highest mortality in sepsis occurs in a subgroup of patients with more than three organ failures and this subgroup is less common than the subgroup with fewer organs failing. Increasing the sample size by enrolling readily available but less severe cases will only increase the proportion of patients with lower mortality. Large “pragmatic” trials are rarely suitable for complex heterogenous conditions like sepsis. Sepsis is not alone in these problems. Other forms of critical illness such as acute respiratory distress syndrome (ARDS) and acute kidney injury (AKI) are similar in terms of clinical and mechanistic heterogeneity and in terms of scarcity of treatment.

The classical approach to interventional clinical trials applied to sepsis and other critical care syndromes has moved confidently from one failure to another. Despite several examples where robust pre-clinical foundations existed and early-stage clinical trials showed promise, phase 3 clinical trials have come up short [3]. Failure at phase 2 or 3 usually spells “certain death” for an investigational agent and yet, given the heterogeneity described above, benefit might still be obtainable for subgroups of patients (for example, those with a specific mechanism of disease) [4]. One potential way forward is to use Bayesian methods in order to incorporate prior experience with an intervention into later-phase clinical trials. Here we explore the advantages of the use of Bayesian statistical methods for clinical trials in the critically ill and we present an example using an intervention for sepsis.

Many authors have advocated for use of Bayesian methods [5–10] citing (a) the flexibility that they can bring to the analyses of complex trials; (b) their ability to incorporate information from outside the current trial; and (c) their ability to better quantify the evidence as to whether a treatment is beneficial. It is beyond the scope of this paper to fully cover the Bayesian approach and we refer the interested reader to the many published articles and key textbooks that present this material to a non-statistical audience [6, 8, 11, 12]. Here, we take the example of a randomized trial of an intervention for sepsis and explain the steps in designing and analyzing the trial based on Bayesian methods. Importantly, while many papers using Bayesian methods in analyses of clinical trials have examined what potential priors do to the interpretation of completed trials [13], our goal here is to design a new trial. As such we have selected a single, empirical source of prior information and examined differences in trial performance according to the method for incorporating this prior information and the choice of analytic method and then present a range of hypothetical results for the new trial analyzed with the proposed methods.

## Methods

The EUPHRATES trial [14] compared 28-day mortality between 223 patients randomized to polymyxin B hemadsorption (PMX) and 226 to sham hemadsorption (control). The trial was performed in accordance with the responsible committee on human experimentation and with the Helsinki Declaration of 1975. Informed consent was obtained from all subjects prior to enrollment. IRB approval Cooper University Hospital (05/18/2010; #09-144). Clinicaltrials.gov [NCT01046669].

Mortality was not significantly different between groups in the intention-to-treat analyses. In a subsequent post hoc analysis [15], where comparisons were restricted to patients completing two treatments and with a Multi-Organ Dysfunction Score (MODS) [16] of 9 or more and endotoxin activity assay (EAA) results between 0.60 and 0.89, and adjusting for baseline APACHE II and mean arterial pressure found an odds ratio (OR) for 28-day mortality of 0.52 (95% CI: 0.27–0.99) in favor of PMX. PMX treatment compared with control also showed greater improvement in MAP (median (IQR) 8 mmHg (− 0.5, 19.5) versus 4 mmHg (− 4.0, 11) *P* = 0.04) and ventilator-free days (median (IQR) 20 days (0.5, 23.5) versus 6 days (0, 20), *P* = 0.004). This subgroup effect is credible since observational studies have found reduced benefit for PMX for patients with lower organ failure scores [17]. Endotoxin activity < 0.6 equates to a burden of endotoxin below the threshold for benefit from PMX in most patients while ≥ 0.9 may identify a population too sick to benefit [15]. These thresholds are not arbitrary because of the mechanism of action of PMX. First, like other forms of blood purification, hemoadsorption relies on concentration-dependent binding; when the solute concentration is lower, removal will be reduced. Second, when solute concentration exceeds an upper limit, the device will no longer be able to achieve an effect and evidence suggests that EAA ≥ 0.9 equates to an endotoxin load beyond the capacity of PMX to impact [18]. Third, using the full EUPHRATES dataset, there was a greater than 97% probability (> 99% in US sites) that the effect of PMX was more beneficial in patients with MODS > 9 and 0.6 ≤ EAA ≤ 0.89 than in the remaining patients. Full details of this subgroup analysis are provided in the supplement. See also *Instrument for assessing the Credibility of Effect Modification Analyses (ICEMAN) in randomized controlled trials* checklist (ICEMAN [19] is provided in the supplement). However, there is interest in confirming the benefit of PMX in this subgroup in a new clinical trial.

A new trial could be performed as a standalone study. However, if analyzed with a chi-squared test of proportions, and assuming the exact 28-day mortality seen in the subgroup (36.7% in PMX and 47.2% in control), it would require 542 patients (271 per arm) to achieve 80% power at a one-side significance level of 0.05. While possible, such a trial would be impractical given that we are selecting a narrow subgroup of the overall septic shock population. Furthermore, the data from EUPHRATES would be put aside when the new data were analyzed. By contrast, an alternative design would be to use Bayesian methods and run the new trial in such a way that the new results could be combined with the prior results from those patients in EUPHRATES with both high MODS and a treatable range of EAA between 0.6 and 0.89 (from now, referred to as the “treatable cohort”) to more efficiently confirm or refute the benefit in such patients. The new trial is called Tigris (NCT03901807)—see supplement. Since the new trial will be performed exclusively in the US, the treatable cohort was further limited to patients from US sites and to achieve greater face validity, the full ITT cohort was used. The final treatable cohort from EUPHRATES was thus 179 patients, 90 PMX/89 control, and unadjusted 28-day mortality was 36.7% versus 47.2%.

The design of Tigris requires that we address two questions. 1. How will the results from the treatable cohort from EUPHRATES [15] be integrated with the results from Tigris? 2. How will the integrated results from these trials be analyzed?

### Data integration across trials

A Bayesian analysis can summarize historical evidence on the size of a treatment effect through what is called the ‘prior distribution’. Although many previous studies provide support for the notion that PMX can reduce mortality [20–23], there are numerous differences between the patient groups in these other studies and the proposed Tigris study, most notably the absence of the EAA biomarker to identify a group most likely to benefit from PMX treatment. The treatment effects from these other studies are not a summary of the evidence for a benefit of PMX in this subgroup, so they cannot be used directly to construct a prior for the treatment effect in Tigris. Furthermore, there are many other differences (e.g., study protocol, patient inclusion criteria, study location, timing of outcomes) that also introduce uncertainty about the applicability of those earlier findings to a new trial. By contrast, when we consider Pocock’s criteria [24] for inclusion of data from historical patients in analysis of a new trial, we find that the treatable cohort [15] from the EUPHRATES study is an ideal source of prior information for the treatment effect in Tigris; standard of care, treatment, patient eligibility, evaluation of outcomes, investigators and ICU locations are largely the same in the two studies. We will demonstrate a range of uses of the EUPHRATES treatable cohort: (a) viewing Tigris as a simple continuation of that cohort, (b) down-weighting the prior evidence it provides or (c) ignoring the results entirely.

**Fig. 1:**
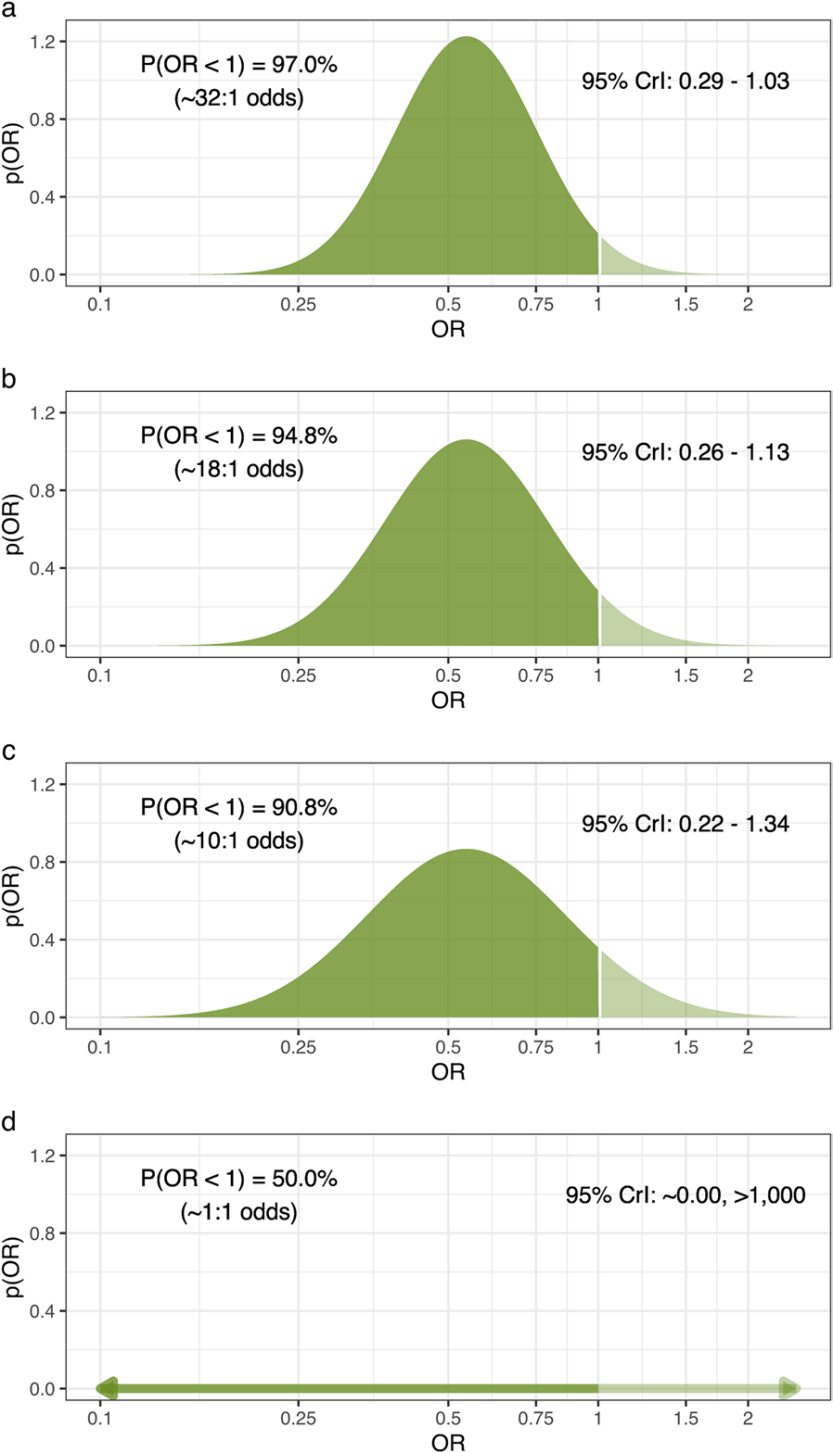
Potential prior distributions for the APACHE-adjusted odds ratio: **a** Prior from the treatable cohort **b** 75% weighted (25% down-weighted) prior from the treatable cohort; **c** 50% down-weighted prior from the treatable cohort; **d** uninformative prior, ignoring external evidence on treatment efficacy, a distribution that is essentially flat over the range of plausible values. Each figure shows the corresponding 95% central credible interval (CrI) and the prior probability that the odds ratio for treatment with PMX is less than 1, along with this same probability expressed as odds in favor of there being a treatment effect (i.e., a 97.0% probability of benefit is the same as an odds of benefit of 97 to 3 or 32.3 to 1)

The extent to which Tigris can be seen as a continuation of enrollment into the treatable cohort from EUPHRATES determines how we use those historical data to create a prior for Tigris. To illustrate this idea, Fig. 1 shows a range of prior distributions formed from the historical APACHE-adjusted odds ratio (aOR) from the treatable cohort. Figure 1a treats Tigris as a straight continuation, and simply takes the posterior distribution of the aOR from the treatable cohort as the prior for Tigris. The priors in Figs. 1b and 1c acknowledge that the previous results may not be entirely transportable to this new trial and are down-weighted to be equivalent to data with the same observed aOR, but in a sample only 75% or 50% of the actual size; these down-weighted priors express more uncertainty than the prior in 1a about the potential values of the aOR. Figure 1d takes an extreme view—the one taken by a classical analysis that uses no prior- ignoring the results in the previous study entirely and allowing a priori that all values of the OR are equally likely, no matter how biologically implausible. The prior appears almost as a horizontal line. Each figure also shows the prior 95% credible interval (CrI), prior probability that the OR for treatment is less than 1 and, because small differences in tail probabilities appear to understate the different levels of certainty in these priors, the corresponding odds that the OR is less than 1.

There are two broad approaches to specifying how much weight should be placed on the historical evidence. One approach uses clinical judgment to fix the weights as shown in Fig. 1 [25] and investigates the results of analyzing new data for each of a small set of fixed weights (e.g., 75%, 100%). The other approach is statistical and uses the similarity between the new data in the trial and the historical data to infer the weight that should be given to the historical evidence; the more similar the new data and the historical data, the higher the weight, and vice versa. We assessed two statistical approaches. The first uses a normalized power prior [26] and the second uses what is called a commensurate prior [27]. Notably, even when the new data are in perfect agreement with the historical data, each of these statistical approaches still places less than full weight on the historical evidence.

A brief summary of the simulations and statistical analyses of the simulations are provided here; full details can be found in the in the supplement. For each of 25 combinations of control group risk (40% to 60% by 5%) and absolute risk reductions (ARR) (0% to 20% by 5%), 2000 trials of 150 patients (100 PMX and 50 control) were simulated. Each trial was analyzed with 5 different uses of the historical data (100% weighting, 75% weighting, weighting through commensurate and normalized power priors, and 0% weighting) with and without adjustment for baseline APACHE score, for a total of 10 analyses per trial. These Bayesian analyses estimated the odds ratio (OR) for mortality comparing the PMX and control groups and in each simulated trial, checked whether the trial demonstrated benefit for PMX, defined in each trial as a posterior probability greater than 95% that the OR was less than 1. The percentage of the 2000 trials demonstrating benefit was used to estimate the power (or type I error when ARR = 0) of the corresponding analytic method for that control group risk and ARR. As a sensitivity analysis, benefit was defined as a posterior probability greater than 97.5% that the OR was less than 1.

When TIGRIS is complete, the trial report will present a plot of the odds ratio and its 95% credible interval against weights ranging from 0 to 100% to allow the reader to assess the dependence of the results on the amount of historical information that is borrowed. We will also present the posterior probability of benefit as a function of these prior weights and will not dichotomize this probability at a sharp threshold of 95%, for example, as being “significant” or not. However, for the purposes of trial planning and investigating the role of the prior, we use these thresholds, an approach that is in keeping with previous literature [28].

## Results

### Effects of baseline risk and prior weighting on power

**Fig. 2:**
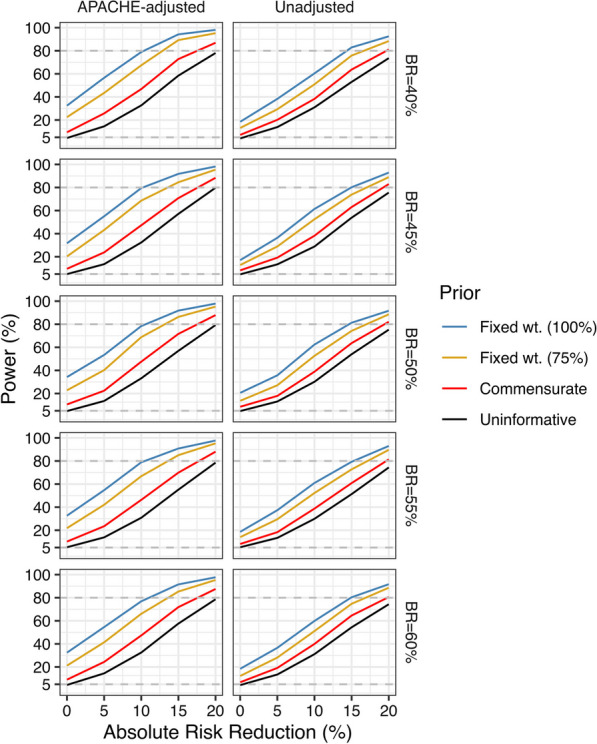
Power (probability of demonstrating benefit at the 95% probability threshold) versus treatment benefit (expressed as the true absolute risk reduction) with APACHE-adjusted and unadjusted analyses for four different uses of the historical data and control group risk of mortality of 40–60%

Each plot in Fig. 2 shows power (the probability that we will conclude that PMX is superior to control at the 95% probability threshold) versus the marginal ARR for treatment. Each plot is for a scenario with the baseline risk shown in the row heading analyzed with either an APACHE-adjusted model (left column) or unadjusted model (right column). Each plot shows power curves versus for fixed 75% and 100% prior weighting, for prior weighting based on similarity of new and historical data using the commensurate prior and for a Bayesian analysis including minimal prior information (uninformative prior). The results shown in Fig. 2 help us make some decisions about the choice of a prior and the analysis, no matter what the true treatment effect and prevalence. There are a few clear patterns. First, an analysis that adjusts for the baseline APACHE II score (left column) is always more powerful than the analysis that does not (right column). Secondly, use of a prior putting 75% weight on the results from the treatable cohort in EUPHRATES (gold lines in each panel in Fig. 2) leads to a greater chance of detecting a true benefit for PMX than a prior that bases the weighting on the similarity of new and historical data (red lines) or an analysis that disregards the historical data (black lines). Thirdly, the increase in power with use of historical data comes with this cost: if there is no true benefit of PMX in Tigris (ARR = 0, far left side of each plot), any analysis that combines a positive signal (from the treatable cohort) with what will be on average a null signal (from Tigris), is more likely to produce a more favorable result than an analysis of Tigris alone [29]. The prior that adjusts the weighting based on the similarity of new and historical data is less likely to produce a favorable result in this null scenario than the fixed 75% weight prior. As the normalized power prior and commensurate prior approaches produced results that were practically identical, only results for the commensurate prior are shown. Additional file 1: Figure S1 presents power when benefit is defined as a posterior probability greater than 97.5% that the OR was less than 1.

The analysis using only Tigris data, as expected, has a 5% chance of meeting the 95% probability threshold for benefit (the black lines in Fig. 2 have “power” of 5% when ARR = 0). When the true ARR in Tigris is 0, some Tigris trial results will vary randomly below an ARR of 0 and, when combined with any use of the historical data, may meet the 95% probability threshold for benefit. Our planned analyses therefore have a small chance of determining that PMX is effective when it is not effective in Tigris. Conversely when the ARR in Tigris is in the neighborhood of 15%, there is still a small chance that we will conclude that PMX is ineffective even though it is highly effective. Still, an analysis combining the historical data with data from the new trial will provide a better representation of the true effect than either the historical data or the new trial data taken separately.

### Potential outcomes for various scenarios

**Fig. 3:**
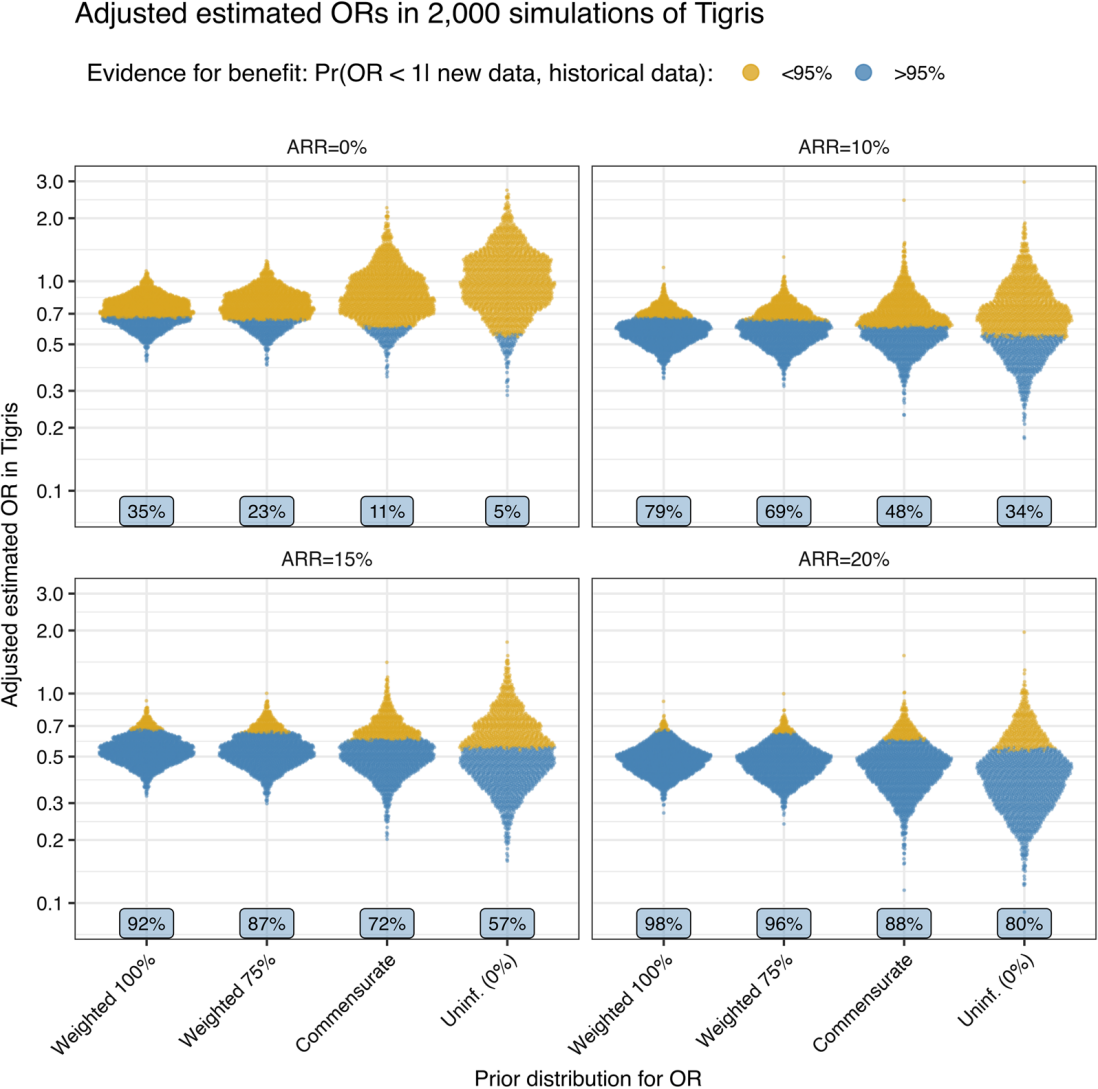
For scenarios with a baseline risk of 50%, distributions across 2000 trials of estimated APACHE-adjusted odds ratios according to the true absolute risk reductions and colored according to whether the Bayesian analysis returns a probability of benefit larger or smaller than 95%. In each panel, each method of analysis (on the x-axis) has the same 2000 trials as input, but more of them lead to a positive finding (colored blue) when more weight is place on the historical evidence. For the planned fixed weighting of 75%, an observed adjusted OR of approximately 0.66 or lower (the threshold separating blue and gold dots) leads to a positive trial conclusion. The blue labels indicate the percentages of simulated trials where we conclude benefit (i.e., the power) for the corresponding absolute risk reduction and use of historical data

Figure 3 provides distributions of observed APACHE-adjusted ORs from the 2000 simulated trials with a baseline risk of 50%, coded according to whether they meet the 95% probability threshold for benefit; a similar plot showing unadjusted ARRs can be found in Additional file 1: Figure S2 and a plot with Bayesian posterior estimated of unadjusted ORs can be found in Additional file 1: Figure S3.

Figures 2 and 3 summarize results over thousands of simulated trials. Table 1 provides a more concrete demonstration of how the different analytic approaches may lead to different conclusions in a single trial. This table shows results of analyses of eight potential trial results, all having an observed control group mortality of 44% (22/50), but with observed mortality in the 100 patients treated with PMX ranging from 24% up to 44%. For observed absolute risk reductions (ARR) from 20% (the first block of rows) and 15% (the second block of rows), Tigris alone would satisfy the criterion of > 95% probability of benefit in both adjusted and unadjusted analyses (cells a and b). The commensurate prior and 75% weighted prior produce still higher probabilities of benefit. In the case of an 11% ARR (33% vs 44%), the adjusted analysis for Tigris alone (cell d in the table), produces only a 92.3% probability of the OR being less than one, a value that rises to 96.1% with the commensurate prior and 98.4% with 75% weight on the prior (cell c). Here, even though the ARR of 11% is almost identical to the 10.5% absolute risk reduction in the treatable cohort, the commensurate prior gives as much credence to the historical data as a 50% weighted prior (result not shown) and returns a probability of benefit of 96.1%, i.e., less than the 97% probability found in the prior. Using the planned 75% weight on the prior, an observed ARR of 7% (37% vs 44%) is approximately the boundary in these eight datasets for reaching the 95% probability threshold for declaring PMX effective. This result gives only a 79.9% probability of benefit in Tigris alone in the adjusted analysis (cell f), but a 95.7% probability when combined with the prior (cell e). In the four potential Tigris trial results that are less favorable (observed ARRs of 5% or less), the posterior probability of benefit in both adjusted and unadjusted analyses fall below the 95% threshold when the prior data are down-weighted to 75%, included through the commensurate prior, or ignored. It may be surprising that a finding of a 1% ARR or even no effect in Tigris (cells g and h) can translate into a posterior probability of benefit of 87%-89% (odds of ~ 7:1 to 8:1) but note that this is lower than the 97% prior probability of benefit (odds 32:1) that we began with; a negative result in Tigris will reduce our belief that PMX is effective. Notably, as an absolute 0% ARR is quite dissimilar to the result in the treatable cohort (10.5% ARR), the commensurate prior gives less credence to the prior data and returns a posterior probability of benefit of 68.3% (cell i).

**Table 1:** Examples of posterior results for potential Tigris outcomes analyzed with different weights on the prior, and with observed PMX absolute risk reductions of 0–20%

Observed mortality in 100 PMX versus 50 control	Use of historical data in prior	Adjusted by APACHE		Unadjusted	
		OR	Pr(OR < 1)	OR	Pr(OR < 1)
24% versus 44% (ARR 20%)	100% fixed weight	0.46 [0.28, 0.76]	100.0	0.53 [0.33, 0.85]	99.5
	75% fixed weight	0.45 [0.27, 0.76]	99.9	0.51 [0.31, 0.84]	99.6
	Commensurate	0.44 [0.21, 0.85]	99.3	0.47 [0.23, 0.84]	99.5
	None (Tigris only)	0.38 [0.16, 0.88]	98.9 (a)	0.40 [0.19, 0.82]	99.3 (a)
29% versus 44% (ARR 15%)	100% fixed weight	0.52 [0.32, 0.84]	99.6	0.59 [0.36, 0.93]	98.8
	75% fixed weight	0.52 [0.31, 0.87]	99.4	0.58 [0.35, 0.96]	98.2
	Commensurate	0.52 [0.28, 0.95]	98.3	0.56 [0.39, 0.98]	97.9
	None (Tigris only)	0.50 [0.24, 1.04]	96.9 (b)	0.52 [0.26, 1.05]	96.6 (b)
33% versus 44% (ARR 11%)	100% fixed weight	0.56 [0.35, 0.92]	99.0	0.64 [0.40, 1.03]	96.8
	75% fixed weight	0.56 [0.34, 0.95]	98.4 (c)	0.63 [0.39, 1.05]	96.2
	Commensurate	0.57 [0.31, 1.07]	96.1	0.64 [0.35, 1.13]	94.0
	None (Tigris only)	0.59 [0.28, 1.22]	92.3 (d)	0.62 [0.31, 1.26]	90.9
37% versus 44% (ARR 7%)	100% fixed weight	0.63 [0.39, 1.00]	97.6	0.69 [0.43, 1.08]	94.7
	75% fixed weight	0.64 [0.38, 1.07]	95.7 (e)	0.70 [0.42, 1.15]	92.1
	Commensurate	0.66 [0.36, 1.28]	89.7	0.71 [0.40, 1.29]	87.5
	None (Tigris only)	0.73 [0.35, 1.56]	79.9 (f)	0.75 [0.37, 1.49]	79.9
39% versus 44% (ARR 5%)	100% fixed weight	0.65 [0.40, 1.05]	96.2	0.72 [0.45, 1.13]	92.1
	75% fixed weight	0.67 [0.40, 1.12]	93.8	0.73 [0.44, 1.20]	89.5
	Commensurate	0.69 [0.39, 1.31]	88.1	0.76 [0.42, 1.39]	83.0
	None (Tigris only)	0.76 [0.37, 1.60]	76.6	0.81 [0.42, 1.63]	72.3
41% versus 44% (ARR 3%)	100% fixed weight	0.66 [0.40, 1.09]	94.8	0.74 [0.47, 1.18]	89.5
	75% fixed weight	0.69 [0.40, 1.17]	91.4	0.76 [0.46, 1.27]	85.5
	Commensurate	0.72 [0.40, 1.41]	83.8	0.80 [0.45, 1.47]	77.2
	None (Tigris only)	0.83 [0.40, 1.72]	69.3	0.88 [0.45, 1.75]	63.5
43% versus 44% (ARR 1%)	100% fixed weight	0.70 [0.43, 1.12]	93.1	0.77 [0.49, 1.22]	86.6
	75% fixed weight	0.73 [0.43, 1.22]	88.7 (g)	0.79 [0.49, 1.29]	82.1
	Commensurate	0.80 [0.45, 1.55]	75.5	0.85 [0.48, 1.60]	70.9
	None (Tigris only)	0.93 [0.46, 1.88]	57.6	0.95 [0.48, 1.92]	55.6
44% versus 44% (ARR 0%)	100% fixed weight	0.72 [0.44, 1.16]	91.0	0.79 [0.50, 1.23]	85.0
	75% fixed weight	0.74 [0.44, 1.26]	86.5 (h)	0.81 [0.49, 1.32]	79.8
	Commensurate	0.85 [0.46, 1.73]	68.3 (i)	0.88 [0.50, 1.68]	66.0
	None (Tigris only)	1.02 [0.49, 2.15]	47.8	1.00 [0.50, 2.00]	49.8

Examples of analyses of potential Tigris results showing observed absolute risk reductions of 0% to 20% from an observed control group event rate of 44% (22/50). Both adjusted and unadjusted analyses are shown, for fixed weights of 100%, 75%, and 0% (analysis of Tigris only) on the prior from the treatable cohort and the commensurate prior. The results show the odds ratio (OR) and 95% CrI, along with the posterior probability that the OR is < 1. Each shaded block of results is based on the same simulated dataset; all data sets share a single set of APACHE II values, with identical sample means and standard deviations in the PMX and control groups. For cell annotations (a–i), see the results section

## Discussion

The synthesis of results from a prior trial into the analysis of a new trial expresses the view that science is engaged in knowledge-building. For example, the totality of what we know about PMX in the target population will be best represented by the synthesized results once Tigris is completed. The use of Bayesian analyses forces an explicit expression of how previous findings will be used in the analysis of new data. Interpretation of results from a standalone trial often involves qualitative comparisons to other trials or observational data, but with no clear message about what the totality of evidence means.

On the weight of existing evidence, there is a weak recommendation against PMX hemadsorption in 2021 Surviving Sepsis Campaign Guideline [30]. Two systematic reviews [22, 23] concluded that hemadsorption in general and hemadsorption with PMX specifically, reduced mortality in patients with sepsis. A third meta-analysis found no benefit from trials with low risk of bias [31]. A propensity score-matched comparison of PMX to non-PMX hemadsorption in 4141 matched pairs found a reduction in all-cause in-hospital mortality with PMX treatment [20]. However, when PMX is used for all patients with sepsis or even septic shock, the overall treatment effect will be attenuated because not all patients will be able to benefit because most do not have endotoxin activity in the target range. Furthermore, not all patients with high endotoxin activity have sufficient organ dysfunction to warrant therapy. Fujimori et al. reported that PMX is not effective when the patients Sequential Organ Failure Assessment (SOFA) scores are < 7 [17]. Thus, using both an organ failure threshold and an EAA range to enrich the patient population ensures that a larger effect size will be achieved. By contrast, most trials in critical illnesses such as sepsis, have used more pragmatic approaches that maximize sample size, on the premise that a larger sample size always increases power of the test of an intervention. The problem is that adding patients who cannot benefit actually reduces power because it lowers the average effect size—it simply isn’t possible to improve trial efficiency by enrolling the wrong patients.

Importantly, the Bayesian analysis we have illustrated is entirely consistent with trials recently conducted to evaluate therapies of COVID-19. In fact, if EUPHRATES had not stopped entirely, but stopped enrollment only of those with EAA ≥ 0.90 and continued to enroll patients with EAA in the 0.60 to 0.89 range with MODS > 9 (an enrichment phase), it would resemble many of the large platform trials that have had so much success recently [32, 33]. If that had been the case, the treatment effect in the EAA-defined subgroup could have been estimated from all the patients who were in the EAA-defined subgroup in both the pre- and post-enrichment phases of the trial. When all the patients are pooled in this way, this is equivalent to forming a prior from the data in the first part of the trial, putting 100% weight on that prior, and updating it the with data from the second part of the trial. Finally, the selection of the treatable cohort from EUPHRATES (i.e., the group used to generate the prior used for Tigris) was not based on statistical “fishing” but rather, is supported by the literature. Observational studies have found reduced benefit for PMX for patients with lower organ failure scores [17], and endotoxin activity 0.9 or higher equates to a burden of endotoxin often beyond the capacity of the device to clear [18]. The ICEMAN instrument [19] provided in the supplement provides a detailed assessment of credibility for effect modification analyses such as this one.

We acknowledge that there will be criticism of our planned analysis because it uses an informative prior to increase the power of the Tigris trial at the expense of an increased frequentist type I error rate. The criticism is that if the true effect of PMX in TIGRIS is exactly zero, our analysis has a greater than 5% chance of concluding benefit for PMX when we use a 95% threshold for concluding benefit. Along with this criticism might come a suggestion that we use a more stringent threshold (e.g., Probability (benefit) > 99%), in order to attain 5% type I error rate. However, it has been shown [29, 34] that use of a more stringent threshold negates any power gains that come from using an informative prior. If data from EUPHRATES are used to create an informative prior favoring treatment, the type I error rate will be greater than (100-P)%, when we set the threshold for declaring benefit at P%. As it is not possible both to have power gains and strict control of type I error with our informative prior, there is no advantage to our use of such a prior if type I error control is of paramount concern. A criticism of power gains made at the expense of an increased type I error rate is a criticism of the use of an informative prior favoring treatment. The trade-offs inherent in the use of prior information are implicit in FDA guidance for the use of Bayesian statistics in medical device trials, [35] which sanctions loosened type I error control with the use of credible prior information: “When using prior information, it may be appropriate to control type I error at a less stringent level than when no prior information is used. For example, if the prior information is favorable, the current trial may not need to provide as much information regarding safety and effectiveness. The degree to which we might relax the type I error control is a case-by-case decision that depends on many factors, primarily the confidence we have in the prior information.” We believe this same logic extends beyond device trials. The question of the importance of type I error (or *p*-values) in study design cannot be resolved here. However, we have presented a study design that transparently adheres to Bayesian principles of data synthesis, along with its frequentist operating characteristics and anticipate healthy debate about our approach when Tigris is complete and analyzed.

## Conclusion

Using Bayesian methods and a biologically credible prior distribution yields a study design with a much smaller sample size than a standalone trial. In our example, when the prior distribution places 75% weight on the historical data, the power for demonstrating benefit at the 95% probability threshold is greater than 80% for ARR of at least 14% in a sample of 150 patients randomized 2:1 in favor of the intervention. Bayesian methods may be a viable option for trials in critical care medicine where treatments have been elusive.

### Supplementary Information

**Additional file 1.** 1.ICEMAN: Instrument for assessing the Credibility of Effect Modification Analyses (ICEMAN) in randomized controlled trials. 2. Detailed Simulation and Statistical Methods. 3. Supplemental Figures and Tables. 4. Tigris Trial Protocol Synopsis. 5. Supplemental references.

## Data Availability

Simulation algorithms and Stan code are available in the supplement. Euphrates source data are available from the sponsor via data use agreement.
