# Correction to: Bayesian methods: a potential path forward for sepsis trials

**DOI:** 10.1186/s13054-023-04791-1

**Published:** 2024-01-03

**Authors:** George Tomlinson, Ali Al-Khafaji, Steven A. Conrad, Faith N. F. Factora, Debra M. Foster, Claude Galphin, Kyle J. Gunnerson, Sobia Khan, Roopa Kohli-Seth, Paul McCarthy, Nikhil K. Meena, Ronald G. Pearl, Jean-Sebastien Rachoin, Ronald Rains, Michael Seneff, Mark Tidswell, Paul M. Walker, John A. Kellum

**Affiliations:** 1 Dalla Lana School of Public Health, University of Toronto, Toronto, ON Canada; 2 Department of Critical Care Medicine, University of Pittsburgh, 3550 Terrace Street, 600 Scaife Hall, Pittsburgh, PA 15261 USA; 3 Departments of Medicine, Emergency Medicine, Pediatrics and Surgery, Louisiana State University Health, Shreveport, LA USA; 4 Department of Intensive Care and Resuscitation, Cleveland Clinic, Cleveland, OH USA; 5 Spectral Medical Inc, Toronto, ON Canada; 6 Southeast Renal Research Institute, CHI Memorial Hospital, Chattanooga, TN USA; 7 Departments of Emergency Medicine, Anesthesiology, and Internal Medicine, University of Michigan Medical Center, Ann Arbor, MI USA; 8 Department of Medicine, Stony Brook University Hospital, Stony Brook, NY USA; 9 Institute for Critical Care Medicine, Icahn School of Medicine at Mount Sinai, New York, NY USA; 10 West Virginia University, Heart & Vascular Institute, Morgantown, WV USA; 11 Division of Pulmonary and Critical Care Medicine, University of Arkansas for Medical Sciences, Little Rock, AR USA; 12 Department of Anesthesiology, Perioperative and Pain Medicine, Stanford University, Stanford, CA USA; 13 Cooper University Healthcare, Cooper Medical School of Rowan University, Camden, NJ USA; 14 Pulmonary Associates, Univ of Colorado Health-Memorial Hospital, Colorado Springs, CO USA; 15 Department of Anesthesia and Critical Care, George Washington University Hospital, Washington, DC USA; 16 Pulmonary and Critical Care Division, Baystate Medical Center, Springfield, MA USA

**Correction to: Crit Care 27, 432 (2023)** 10.1186/s13054-023-04717-x

Following publication of the original article [1], the authors identified errors in the Additional file 1. The Figures on pages 15, 16 of the Additional file 1 (S2 and S3) have been corrected and some minor corrections to the table of contents were made.

The correct Additional file 1 file is included in this article.

Additional file 1 has been updated in this correction article and the original article [1] has been corrected.

### Supplementary Information

**Additional file 1.** 1. ICEMAN: Instrument for assessing the Credibility of Effect Modification Analyses (ICEMAN) in randomized controlled trials. 2. Detailed Simulation and Statistical Methods. 3. Supplemental Figures and Tables. 4. Tigris Trial Protocol Synopsis. 5. Supplemental references.
