# Supplementary material for: Bayesian methods: a potential path forward for sepsis trials
Source: Crit Care. 2023 Nov 8;27:432. doi: 10.1186/s13054-023-04717-x (PMC10634134; doi:10.1186/s13054-023-04717-x)
Supplement: Supplementary file 1 — Additional file 1. 1.ICEMAN: Instrument for assessing the Credibility of Effect Modification Analyses (ICEMAN) in randomized controlled trials. 2. Detailed Simulation and Statistical Methods. 3. Supplemental Figures and Tables. 4. Tigris Trial Protocol Synopsis. 5. Supplemental references. [file 13054_2023_4717_MOESM1_ESM.pdf]

Bayesian methods: A potential path forward for sepsis trials.

Supplemental Material

Contents

1	Instrument for assessing the Credibility of Effect Modification Analyses (ICEMAN) in randomized controlled trials<br>*a. ICEMAN worksheet*<br>*b. Interaction of subgroup and PMX effect in EUPHRATES*	2-4<br>*2-3*<br>*4*
2	Detailed Simulation and Statistic Methods<br>*a. EUPHRATES Trial*<br>*b. Statistical model for Tigris*<br>*c. Algorithm for simulation study*<br>*d. Stan code supplement*	5-14<br>*5*<br>*5-6*<br>*7-10*<br>*10-13*
3	Supplemental Figures and Tables<br>Figure S1<br>Figure S2<br>Figure S3	14-16<br>*14*<br>*15*<br>*16*
4	Tigris Trial Protocol Synopsis	17-22
5	Supplemental references	23

1. ICEMAN: Instrument for assessing the Credibility of Effect Modification Analyses (ICEMAN) in randomized controlled trials *Version 1.0*

Quick instructions

- Synonyms for effect modification include subgroup effect, interaction, and moderation
- The instrument applies to a single proposed effect modification at a time; complete one form per each outcome, time-point, effect measure, and effect modifier
- Response options on the left indicate definitely or probably reduced, response options on the right probably or definitely increased credibility
- Completely unclear goes under probably reduced credibility
- It is helpful to provide a supporting comment or quotation under each question
- Whether an effect modification is patient-important is not part of the credibility assessment
- The manual provides more detailed instructions and examples

Preliminary considerations

Study reference(s):

If available, protocol reference(s): **See supplement section 4**

State a single outcome and, if applicable, time-point of interest (e.g., mortality at 1 year follow-up): **28-day mortality**

State a single effect measure of interest (e.g., relative or absolute risk difference): **Odds Ratio**

State a single potential effect modifier of interest (e.g., age or comorbidity): **Endotoxin activity (EAA)**

Was the potential effect modifier measured before or at randomization? [**X**] yes, continue [ ] no, stop here, refer to manual for further instructions

Credibility assessment

1: Was the direction of the effect modification correctly hypothesized a priori?

[ ] Definitely no	[ ] Probably no or unclear	[ ] Probably yes	[**X**] Definitely yes
Clearly post-hoc or results inconsistent with hypothesized direction or biologically very implausible	*Vague hypothesis or hypothesized direction unclear*	*No prior protocol available but unequivocal statement of a priori hypothesis with correct direction of effect modification*	*Prior protocol available and includes correct specification of direction of effect modification, e.g., based on a biologic rationale*

Comment:

2: Was the effect modification supported by prior evidence?

[ ] Inconsistent with prior evidence	[ ] Little or no support or unclear	[**X**] Some support	[ ] Strong support
Prior evidence suggested a different direction of effect modification	*No prior evidence or consistent with weak or very indirect prior evidence (e.g., animal study at high risk of bias) or unclear*	*Consistent with more limited or indirect prior evidence (e.g., large observational study, non-significant effect modification in prior RCT, or different population)*	*Consistent with strong prior evidence directly applicable to the clinical scenario (e.g., significant effect modification in related RCT)*

Comment: A laboratory study by Romaschin et al.[1] is cited for justification of the analysis in the subgroup with EAA < 0.9. Romaschin writes "the results presented in this this study suggest that the adsorption capacity of PMX-20R is sufficient to remove a clinically significant amount of endotoxin in a majority of endotoxemic septic shock patients; however, this may not be the case in patients with a high EAA burden >0.9." Furthermore, results from a recent registry study found that patients with EAA between 0.6-0.9 and treated with PMX had similar outcomes to those in the EUPHRATES treatable cohort.[2]

3: Does a test for interaction suggest that chance is an unlikely explanation of the apparent effect modification? (consider irrespective of number of effect modifiers)

[ ] Chance a very likely explanation	[ ] Chance a likely explanation or unclear	[**X**] Chance may not explain	[ ] Chance an unlikely explanation
Interaction p-value >0.05	*Interaction p-value ≤0.05 and >0.01, or no test of interaction reported and not computable*	*Interaction p-value ≤0.01 and >0.005*	*Interaction p-value ≤0.005*

Comment: The initial EUPHRATES trial paper did not carry out a test of interaction with the specific subgroup of interest. The Klein paper presents results only on that subgroup, so no test of interaction is available. However, we have reanalyzed the EUPHRATES data (see 1b below) and found strong evidence for interaction especially for the specific prior being used—MODS >9, EAA between 0.6-0.9, US sites. The Bayesian analysis does not produce a p-value, but when defining the EUPHRATES subgroup to match the Tigris study entry criteria, there was a greater than 99% probability (99.3% in unadjusted analysis and 99.6% in APACHE-II-adjusted analysis) that the treatment effect was larger in this treatable US-based cohort than in the remaining US patients. These posterior probabilities correspond approximately to one-side p-values of 0.007 and 0.004, so we have picked the ICEMAN response that includes both, even if they are doubled to get two-sided p-values.

4: Did the authors test only a small number of effect modifiers or consider the number in their statistical analysis?

[ ] Definitely no	[ ] Probably no or unclear	[ ] Probably yes	[**X**] Definitely yes
Explicitly exploratory analysis or large number of effect modifiers tested (e.g., greater than 10) and multiplicity not considered in analysis	*No mention of number or 4-10 effect modifiers tested and number not considered in analysis*	*No protocol available but unequivocal statement of 3 or fewer effect modifiers tested*	*Protocol available and 3 or fewer effect modifiers tested or number considered in analysis*

Comment: Only the high-MODS, EAS 0.6-0.89 subgroup was assessed

---

5: If the effect modifier is a continuous variable, were arbitrary cut points avoided? [ ] not applicable: not continuous

[ ] Definitely no	[ ] Probably no or unclear	[**X**] Probably yes	[ ] Definitely yes
Analysis based on exploratory cut point (e.g., picking cut point associated with highest interaction p-value)	*Analysis based on cut point(s) of unclear origin*	*Analysis based on pre-specified cut points, e.g., suggested by prior RCT*	*Analysis based on the full continuum, e.g., assuming a linear or logarithmic relationship*

Comment: The Romaschin study[1] defines the upper limit of treatability with standard PMX treatment. In the EUPHRATES RCT, a protocol amendment after the second interim analysis (after advice from the DSMC and the FDA) restricted further enrollment to those with MODS of > 9; in total 80% of the 162 deaths occurred in the 43% of participants with MODS > 9 (where mortality was ~ 45%). In the smaller group with MODS < 9, there were 32 deaths in 154 participants (21% mortality). That MODS cut-point was based on the overall risk-benefit assessment, not a differential effect of PMX.

---

6 Optional: Are there any additional considerations that may increase or decrease credibility? (manual section 2.6)

[ ] Yes, probably decrease [**X**] Yes, probably increase

Comment: A prior study by Marshall et al.[3] found that endotoxin activity below 0.6 units by EAA identified patients at lower risk of death and therefor identifies a population unlikely to benefit from endotoxin removal. Conversely extracorporeal blood purification devices have an upper limit with respect to removal capacity before reaching saturation. Result from Romaschin et al.[1] indicate that EAA >0.9 corresponds to the likely upper limit of treatabilty with a standard course of PMX hemadsorption. Finally, observational studies have found reduced benefit for PMX for patients with lower organ failure scores.[4]

7: How would you rate the overall credibility of the proposed effect modification?

The overall rating should be driven by the items that decrease credibility. The following provides a sensible strategy:

- All responses definitely or probably reduced credibility or unclear → very low
- Two or more responses definitely reduced credibility → maximum usually low even if all other responses satisfy credibility criteria
- One response definitely reduced credibility → maximum usually moderate even if all other responses satisfy credibility criteria
- Two responses probably reduced credibility → maximum usually moderate even if all other responses satisfy credibility criteria
- No response options definitely or probably reduced credibility → high very likely
- Place a mark on the continuous line (or type "x" in electronic version)

			**X**
**Very low credibility**	**Low credibility**	**Moderate credibility**	**High credibility**
Very likely no effect modification<br>Use overall effect for each subgroup	Likely no effect modification<br>Use overall effect for each subgroup but note remaining uncertainty	Likely effect modification<br>Use separate effects for each subgroup but note remaining uncertainty	Very likely effect modification<br>Use separate effects for each subgroup

b. Interaction of subgroup and PMX in EUPHRATES

The subgroup is defined by MODS > 9 AND 0.6 ≤ EAA ≤ 0.89. The reference group is everyone not in the subgroup.

The counts of observations cross-classified by treatment, subgroup and country are shown below. We check for an interaction of treatment and subgroup in all EUPHRATES patients and, because Tigris is being run only in the USA, in the EUPHRATES patients at sites in the USA.

	Canada EUPHRATES		USA EUPHRATES		All EUPHRATES	
Treatment	Reference Group	Subgroup	Reference Group	Subgroup	Reference Group	Subgroup
Control	26	31	80	89	106	120
PMX	30	23	81	90	111	113
Total	56	54	161	179	217	233

<u>Models</u>

Unadjusted

$$\log(\text{odds}(\text{28-d mortality})) = a + b_1{*}G + b_2{*}PMX + b_3{*}G{*}PMX$$

Adjusted

$$\log(\text{odds}(\text{28-d mortality})) = a + b_1{*}G + b_2{*}PMX + b_3{*}G{*}PMX + b_4{*}APACHE$$

G = 1 when MODS > 9 AND 0.6 ≤ EAA < 0.9
= 0 otherwise
PMX = 1 when treatment = PMX
= 0 otherwise

In this model, b_3 is the difference in log odds ratios between the subgroup and the reference group, and $\exp(b_3)$ is the interaction effect, the ratio of the odds ratio in the subgroup (moderate EAA and MODS > 9) to the odds ratio in the reference group (EAA < 0.6 or EAA > 0.89 or MODS ≤9)

<u>Results</u>

Analysis set	Adjusted by Apache II?	Interaction effect [exp(b3)] Posterior Mean and 95% CrI	Posterior Pr(Interaction effect <1)
All EUPHRATES (USA and Canada)	No	0.51 [0.22, 1.02]	97.1%
	Yes	0.48 [0.19, 0.99]	97.6%
USA EUPHRATES	No	0.36 [0.13, 0.81]	99.3%
	Yes	0.32 [0.11 ,0.73]	99.6%

2. Detailed Simulation and Statistical Methods

a. The EUPHRATES Trial

EUPHRATES, "Evaluating the Use of Polymyxin B Hemoperfusion in a Randomized Controlled Trial of Adults Treated for Endotoxemia and Septic Shock" was a multicenter, randomized, clinical trial.[5] Study procedures were performed in accordance with the ethical standards of the responsible committee on human experimentation (institutional or regional) and with the Helsinki Declaration of 1975. Prior to randomization, informed consent was obtained from all subjects or their surrogates based on meeting all of the eligibility criteria. The first institutional IRB approval was obtained on 05/18/2010 from Cooper University Hospital IRB (#09-144). The study is registered with clinicaltrials.gov as [NCT01046669].

b. Statistical model for Tigris

The binary outcome of 28-day mortality will be compared between groups using logistic regression. This allows prior information about the treatment effect to be included as an OR and also gives an option of including pre-specified covariates related to the outcome, which increases the chance of showing that a treatment is beneficial in a randomized trial.[6-8] However, a more conservative unadjusted analysis may still be preferable assuming that randomization is effective.

The result of the analysis that updates the prior distribution of the OR with new data from Tigris is the posterior distribution of the OR; this pools the evidence from both the prior and the trial. When Tigris is complete, we will use the posterior distribution for the OR for PMX to compute the probability that PMX is effective, P(OR < 1). With those results in hand, there will be no reason to use a fixed threshold for what is considered strong evidence versus less strong evidence (for example, interpreting a 94.9% probability of benefit as being not strong evidence and a 95.1% probability of benefit as strong evidence). But when designing a Bayesian study, it can be useful to calculate the chance that a trial will find probabilities of benefit larger than key thresholds (e.g., > 97.5%, >95%) under various scenarios defined by the risk of mortality and the true effect of PMX in Tigris. These evaluations allow us to decide if the planned sample size is adequate and to identify the prior with the most desirable properties.

To help decide between a few combinations of analyses and priors in the design for Tigris, we ran a set of simulated trials. Given the benefits of PMX seen in EUPHRATES, for ethical reasons and to encourage enrollment, the randomization ratio will be 2:1 in favour of PMX. We chose a sample size of 150 subjects based on simulations performed using an effect size of 10% - 15% and a down-weighting of the prior to 75% with a 95% threshold probability for declaring PMX superior to control and with this sample size, investigate other prior weights and analytic approaches. A larger sample size would be required if the

prior is down-weighted more or if the effect size is less than the estimate. Trials were simulated using logistic regression with a baseline APACHE II score as a covariate.

$$\text{logit}(p) = \alpha + \beta_1 \times (APACHEII) + \beta_2 \times TMT$$

where p is the risk of mortality for a patient with a known baseline APACHE II score and treatment group (TMT=1 for PMX and 0 for control). Simulations varied the control mortality risk by changing α and the true treatment effect by varying β_2, the log OR for treatment with PMX. The value of β_1 (the log OR for a 1-point increase in APACHE II) was fixed at its value in the EUPHRATES treatable cohort.

Combinations of true mortality risk and treatment effect: we simulated 2,000 trials at each of the 25 combinations of

a. the true control group 28-day risk of mortality in Tigris (40% to 60% by 5%)
b. the true marginal absolute risk reductions (ARRs) of (0% to 20% by 5%)

Options for priors and analysis: every trial was analyzed with each combination of

a. five approaches to incorporating prior information from historical data from the treatable cohort: use of fixed weights of 100%, 75%, and 0% on the prior, use of a normalized power prior, and use of a commensurate prior
b. two analytic models: an unadjusted analysis and an analysis adjusted by APACHE-II
c. two threshold probabilities for declaring PMX superior to control: 97.5% and 95%

In each analysis, we tested whether PMX was superior to control at each threshold probability and, for each combination, analysis, and prior weight, we calculated the percentage of the 2,000 trials in which we concluded PMX was superior to control. After viewing these results, the choice of the prior for the primary analysis of Tigris was based partly on judgment about the similarity of the Tigris trial patients and the treatable cohort from EUPHRATES and partly on the probability of concluding benefit. In classical analysis, this probability is called power when ARR > 0% and it is called type I error when ARR=0%.

Finally, to illustrate how the Bayesian analyses of Tigris will proceed with the chosen prior and threshold probability once we have a single trial result, we present results for hypothetical data exemplifying three groups of scenarios: (1) an observed treatment effect in Tigris of a similar magnitude to that observed in treatable cohort from EUPHRATES (7%-11% absolute risk reduction); (2) observed treatment effects in Tigris suggesting either no benefit or only a small amount of benefit (absolute risk reductions of 0-5%); and (3) observed treatment effects that are greater in magnitude to that seen in the treatable cohort from EUPHRATES (absolute risk reductions of >15%).

c. Algorithm for simulation study

1. **Estimate 3 key parameters from the treatable cohort**:
   a. With 28-day mortality as the outcome, fit a frequentist logistic regression model to estimate these two parameters
      - The log-odds ratio for a one-unit change of APACHE score. This value is used in the simulation of trial data.
      - The APACHE-adjusted log-odds ratio comparing the PMX-treated and control groups. The maximum likelihood estimate and its estimated standard error are used to construct prior distributions for APACHE-adjusted analysis of the simulated trials (in item number 2 below).

   b. With 28-day mortality as the outcome, fit an unadjusted frequentist logistic regression model in the treated cohort to estimate the unadjusted log-odds ratio comparing the PMX-treated and control groups. The maximum likelihood estimate and its estimated standard error are used to construct prior distributions for unadjusted analysis of the simulated trials (in item number 2 below).

   Models for both unadjusted and adjusted analyses of the treatable cohort were fitted in R using the glm function.

2. **Create prior distributions for the log-odds ratios for treatment:** Use the results of the modelling in steps 1a and 1b to create a "base" prior for the APACHE-adjusted and unadjusted log-odds ratios for treatment. The priors were approximated by normal distributions, as the likelihoods from step 1 were close to normal.
   i. $\log(OR_{adjusted})$~ N(mean=-0.605, SD=0.337)
   ii. $\log(OR_{unadjusted})$~ N(mean=-0.435, SD=0.314)

   Results from two classes of priors formed from these base priors are presented in the simulation study. The normalized power prior can be seen as putting a prior on the weight W, but as we found the results from this prior were very similar to results from the commensurate prior, we provide no further details here.

   (a) A prior with a specified fixed weight on the base prior, where the weight is selected from the range 0 < W ≤ 1:

   $\log(\mathrm{OR}_{\mathrm{adjusted}})$ ~ N(mean=-0.605,SD=0.337/$\sqrt{W}$)
   $\log(OR_{\mathrm{unadjusted}})$ ~ N(mean=-0.435,SD=0.314/$\sqrt{W}$)

   (b) A commensurate prior that allows the data to "tell us" how much to inflate the base prior standard deviation. These introduce an additional parameter SD_{comm}.

   $\log(\mathrm{OR}_{\mathrm{adjusted}})$~N(mean=-0.605,SD=$\sqrt{0.337^2 + SD_{comm}^2}$)
   $\log(\mathrm{OR}_{\mathrm{unadjusted}})$~N(mean=-0.435,SD=$\sqrt{0.314^2 + SD_{comm}^2}$)

   In each case, the prior for the additional parameter is the half-normal:

   $\mathrm{SD}_{\mathrm{comm}} = abs(Z)$, where $Z \sim$ N(mean=0,SD=1)

3. **Find the values of $\boldsymbol{\alpha}$, $\boldsymbol{\beta_1}$, and $\boldsymbol{\beta_2}$** in the logistic regression model below that give a true control group mortality risk of **p** and a true absolute risk reduction between PMX and control of **d:**

$$\mathrm{logit}(p(Y|x)) = \alpha + \beta_1 \times x + \beta_2 \times TMT$$

where Y = binary mortality outcome, x = APACHE II, and TMT = binary treatment indicator

Distribution of x: The values of x are assumed to come from a normal distribution with the observed mean APACHE (m) and standard deviation (s) from the treatable cohort. That is x ~ N(x| m, SD) , where N() here is used to denote the normal density function.

Value of β_1.The posterior mean of the log(odds ratio) for APACHE from step (1a) is used as the value for β_1.

Values of α and β_2. The steps below were used to solve for the values of α and β_2 that give the required true control group risk **p** and absolute risk reduction **d**.

a. In the control group, the mortality risk **p**=p(Y=1|x,TMT=0) for a control group patient with a given value of x is related to x by the equation below.

$$\text{p(Y=1| x, TMT=0)} = \frac{1}{1+\exp(-\alpha - \beta_1 x)}$$

b. The marginal risk **p** in a control group with x distributed according to the density N(x|m,s) is given by integrating p(Y=1|x,TMT=0) over the density of x:

$$\boldsymbol{p} = p(Y=1|TMT=0) = \int (1/(1+\exp(-\alpha - \beta_1 x)))\, N(x,|m,s)dx$$

A grid search was used to find the value of α satisfying the equation above for known **p**, β_1, m, and s

c. The marginal risk of mortality in the PMX group, **p-d**, is given by integrating p(Y=1|x, TMT=1) over the distribution of x:

$$\boldsymbol{p} - \boldsymbol{d} = p(Y=1|TMT=1) = \int (1/(1+\exp(-\alpha - \beta_1 x - \beta_2)))N(x,|m,s)dx$$

With α from (3b) and β_1 from (1a), a grid search was used to find the value of β_2 giving PMX true group mortality risk of **p-d** for known **p**,**d**, β_1, m, and s . This gives the fully specified logistic regression model for simulating Y corresponding to a baseline risk **p** and ARR **d**.

$$\text{logit}(p(Y|x,TMT)) = \alpha + \beta_1 \times x + \beta_2 \times TMT$$

4. **Simulate a single data set:** For a given **p** and **d,** which determine α and β_2, simulate a data set with 150 observations.

   a. Generate 150 independent random values APACHE_i from N(m, s), i=1,150

   b. Assign the first 100 to PMX (TMT_i=1) and the next 50 to control (TMT_i=0)

   c. For each of the 150 (APACHE_i, TMT_i) pairs, use the equation below to generate a mortality risk

   $$p_i = 1/(1+\exp(-\alpha - \beta_1\, APACHE_i - \beta_{TMT} TMT_i))$$

   d. Simulate 150 random uniform variables U_i and set Y_i = 1 if $U_i < p_i$ and 0 otherwise.

   e. The dataset comprises the 150 sets of the triplets (Y_i, APACHE_i, TMT_i)

5. **Analyze the simulated dataset:** Analyze the dataset created in (4) with 8 different Bayesian models (ignoring the power prior):

   I. Unadjusted analysis with uninformative prior for the *unadjusted* log-odds ratio of treatment

II. Unadjusted analysis with a fixed 75% weight (W=0.75) on the base prior for the *unadjusted* log-odds ratio of treatment

III. Unadjusted analysis with a commensurate prior for the unadjusted log-odds ratio of treatment

IV. Unadjusted analysis with a fixed 100% weight (W=1.00) on the base prior for the *unadjusted* log-odds ratio of treatment

V. APACHE-adjusted analysis with uninformative prior for the *adjusted* log-odds ratio of treatment

VI. APACHE-adjusted analysis with a fixed 75% weight (W=0.75) on the base prior for the *adjusted* log-odds ratio of treatment

VII. APACHE-adjusted analysis with a commensurate prior for the *adjusted* log-odds ratio of treatment

VIII. APACHE-adjusted analysis with a fixed 100% weight (W=1.00) on the base prior for the *adjusted* log-odds ratio of treatment

In each of these models, the parameters α and β_1 were given uninformative priors and an additional pre-EUPHRATES N(0, SD=1.175) prior was included for the treatment effect β_2 that put 95% probability on values in the range 0.1 to 10 for the odds ratio for treatment.

For each of the 8 models, save the resulting model fits and calculate, from the MCMC samples

a) Posterior means and 95% credible intervals for the log-odds ratios for treatment
b) The proportion of values of the posterior samples of log-odds ratios for treatment that are below zero (a value that estimates the posterior probability of benefit.)
c) 4 binary variables indicating whether the proportion in (b) is larger than 0.95 and 0.975
    a. I_{95} = I[P(logOR|$data_{sim}$) > 0.95]
    b. $I_{97.5}$ = I[P(logOR|$data_{sim}$) > 0.975]

6. **Simulate multiple trials at combinations of control group risk and marginal risk difference and save the results**

For true control group 28-day risk of mortality **p** in (40% to 60% by 5%){

For true absolute risk reduction with PMX **d** in (0% to 20% by 5%){

Find the values of α and β_2 corresponding to **p** and **d** (using (3))

Simulate 2,000 trials (using (4))

Analyze each trial with 8 different models (using (5)) and save the results

For each model, Calculate the proportion of the 2,000 trials satisfying the criteria I_{95} and $I_{97.5}$

}

}

The proportions satisfying criterion I_{95} and $I_{97.5}$ are used to estimate the probability of trial success for the corresponding combination of **p**, **d** and the analytic method.

All simulations were run in R, using the rstan and brms packages. Four parallel chains were run for each model fit, with 1,000 warm-up iterations and a further 5,000 iterations on which MCMC samples were saved. Convergence to a stationary distribution with this number of iterations was checked on a subset of the simulated datasets, and as expected, given the simplicity of these logistic regression models with sufficient events, models converged quickly.

d.   Stan code supplement

# Model 1: Minimally informative prior, unadjusted

```
data {

  int<lower=0>          ysum[2];
  int<lower=0>          nsum[2];
  int<lower=0>          groupsum[2];
  real<lower=0>         sd00 ;
  real                  mu00;

}
parameters {
  real alpha;
  real beta0;

}

model {
  // initial prior
  target += normal_lpdf(beta0 | mu00, sd00);

  for(k in 1:2)
      target += binomial_logit_lpmf(ysum[k]| nsum[k],
                                    alpha + beta0*groupsum[k]);


}
```

# Model 2: Minimally informative prior, adjusted

```
data {
  int<lower=0>          n ;
  int<lower=0,upper=1>  y[n];
  vector[n]             group;
  vector[n]             apache;
  real<lower=0>         sd00 ;
  real                  mu00;
}

parameters {
  real alpha;
  real beta0;
  real beta_apache;

}
```

```
model {
  target += normal_lpdf(beta0 | mu00, sd00) ;

  target += bernoulli_logit_lpmf(y|
                        alpha + beta0 * group + beta_apache * apache) ;


}
```

# Model 3: Fixed weight, power-prior unadjusted

```
data {

  int<lower=0>         ysum[2];
  int<lower=0>         nsum[2];
  int<lower=0>         groupsum[2];

  //original prior for  historical data
  real<lower=0>        sd00 ;
  real                 mu00;

  //parameterizing approximate normal likelihood of historical data
  real<lower=0>        sd0 ;
  real                 mu0;

//fixed value of w in fixed weight model
  real<lower=0>        w;
}
parameters {
  real alpha;
  real beta0;

}

model {
  // initial prior
  target += normal_lpdf(beta0 | mu00, sd00)  ;
  // power of historical likelihood
  target += w*normal_lpdf(beta0 | mu0, sd0)  ;
  // current likelihood
  for(k in 1:2)
  target += binomial_logit_lpmf(ysum[k]| nsum[k],
                                        alpha + beta0*groupsum[k]) ;

}
```

# Model 4: Fixed weight power, adjusted

```
data {
  int<lower=0>         n ;
  int<lower=0,upper=1> y[n];
  vector[n]            group;
  vector[n]            apache;

  //original prior for  historical data
  real<lower=0>        sd00 ;
  real                 mu00;
```

```
  //parameterizing approximate normal likelihood
  //of historical data
  real<lower=0>        sd0_adj ;
  real                 mu0_adj;
  //fixed value of w in fixed model
  real<lower=0>        w;
}
parameters {
  real alpha;
  real beta0;
  real beta_apache;

}

model {
  // initial prior
  target += normal_lpdf(beta0 | mu00, sd00)  ;

  // power of historical likelihood
  target += w*normal_lpdf(beta0 | mu0_adj, sd0_adj)  ;

  // current likelihood
  target += bernoulli_logit_lpmf(y|
                        alpha + beta0 * group + beta_apache*apache);


}
```

# Model 5: Commensurate prior, unadjusted

```
data {
  int<lower=0>         n ;
  int<lower=0,upper=1> y[n];
  vector[n]            group;

  //original prior for  historical data
  real<lower=0>        sd00 ;
  real                 mu00;

  //parameterizing approximate normal likelihood
  // of historical data
  real<lower=0>        sd0 ;
  real                 mu0;

  //commensurate prior sd
  real<lower=0>  sd_comm_sd ;
}
parameters {
  real alpha;
  real beta0;
  real<lower=0> sd_comm;

}
model {
// original prior
```

```
  target += normal_lpdf(beta0| mu00, sd00)    ;

  // location commensurate prior for beta0
  target += normal_lpdf(beta0 |
                              mu0, sqrt(square(sd0)+square(sd_comm));

  // prior for commensurability parameter
  // (half normal with SD = sd_comm_sd)
  target +=normal_lpdf(sd_comm | 0, sd_comm_sd)  ;

  // current likelihood
  target += bernoulli_logit_lpmf(y| alpha + beta0*group) ;

}
```

# Model 6: Commensurate prior, adjusted

```
data {
  int<lower=0>         n ;
  int<lower=0,upper=1> y[n];
  vector[n]            group;
  vector[n]            apache;

  //original prior for  historical data
  real<lower=0>        sd00 ;
  real                 mu00;

  //parameterizing approximate normal likelihood
  //of historical data

  real<lower=0>        sd0_adj ;
  real                 mu0_adj;
  // commensurate prior sd
  real<lower=0>  sd_comm_sd ;
}
parameters {
  real alpha;
  real beta0;
  real beta_apache ;
  real<lower=0> sd_comm;

}
model {
// original prior
  target += normal_lpdf(beta0| mu00, sd00)    ;

  // location commensurate prior for beta0
  target += normal_lpdf(beta0 | mu0_adj,
                              sqrt(square(sd0_adj)+square(sd_comm)));
  // prior for commensurability parameter
  // (half normal with SD = sd_comm_sd)
  target +=normal_lpdf(sd_comm | 0, sd_comm_sd)  ;

  // current likelihood
  target += bernoulli_logit_lpmf(y|
                        alpha + beta0*group + beta_apache*apache);

  }
```

3. Supplemental figures

Figure S1: Power (probability of demonstrating benefit at the 97.5% probability threshold) versus treatment benefit (expressed as the true absolute risk reduction) with APACHE-adjusted and unadjusted analyses for four different uses of the historical data and control group risk of mortality (BR) of 40% to 60%.

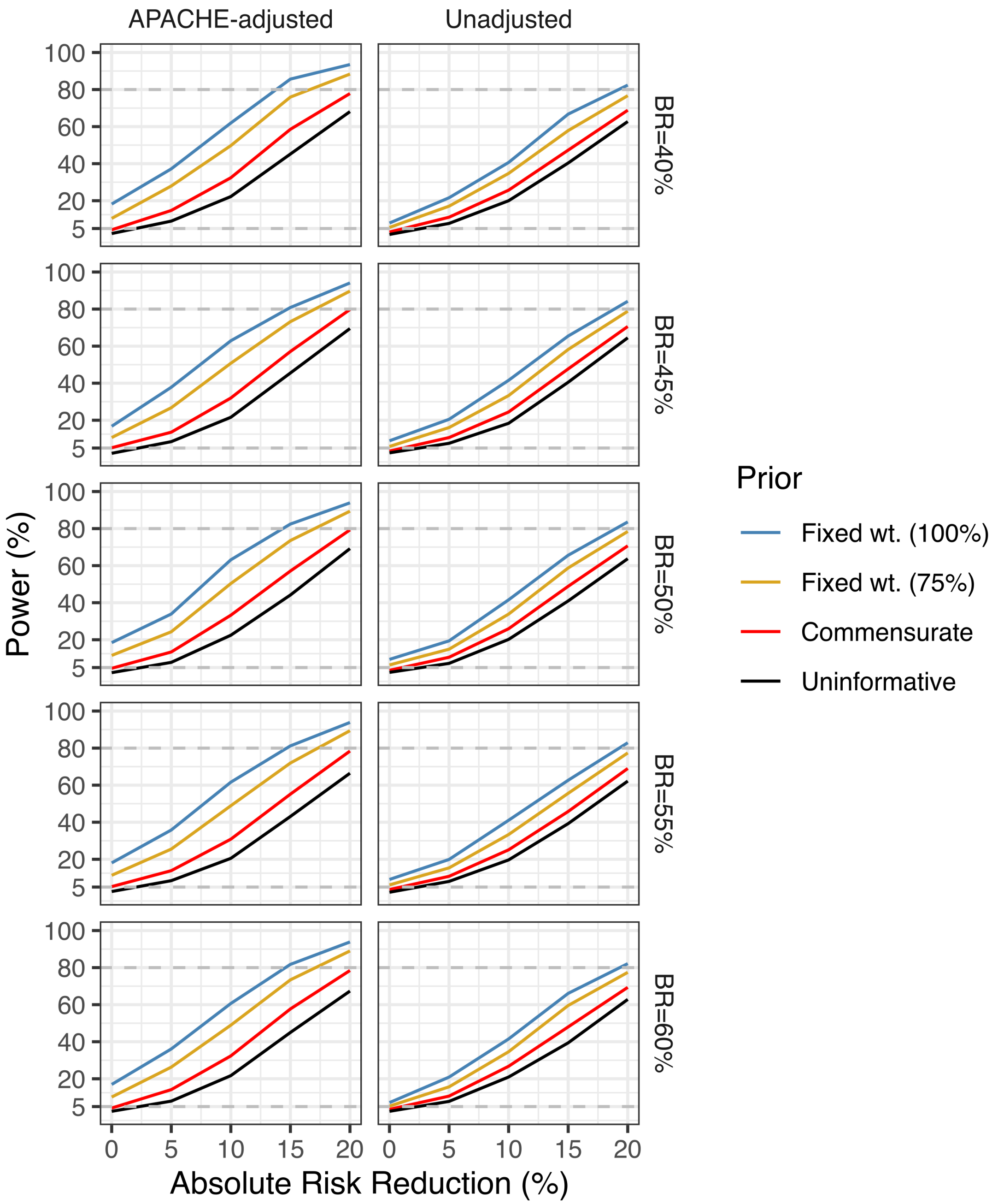

Figure S2: Unadjusted absolute risk reductions in 2,000 simulated trials with a baseline risk of 50%. Blue labels indicate the percentages of simulated trials where we conclude benefit (i.e., the power) for the corresponding absolute risk reduction and use of historical data.

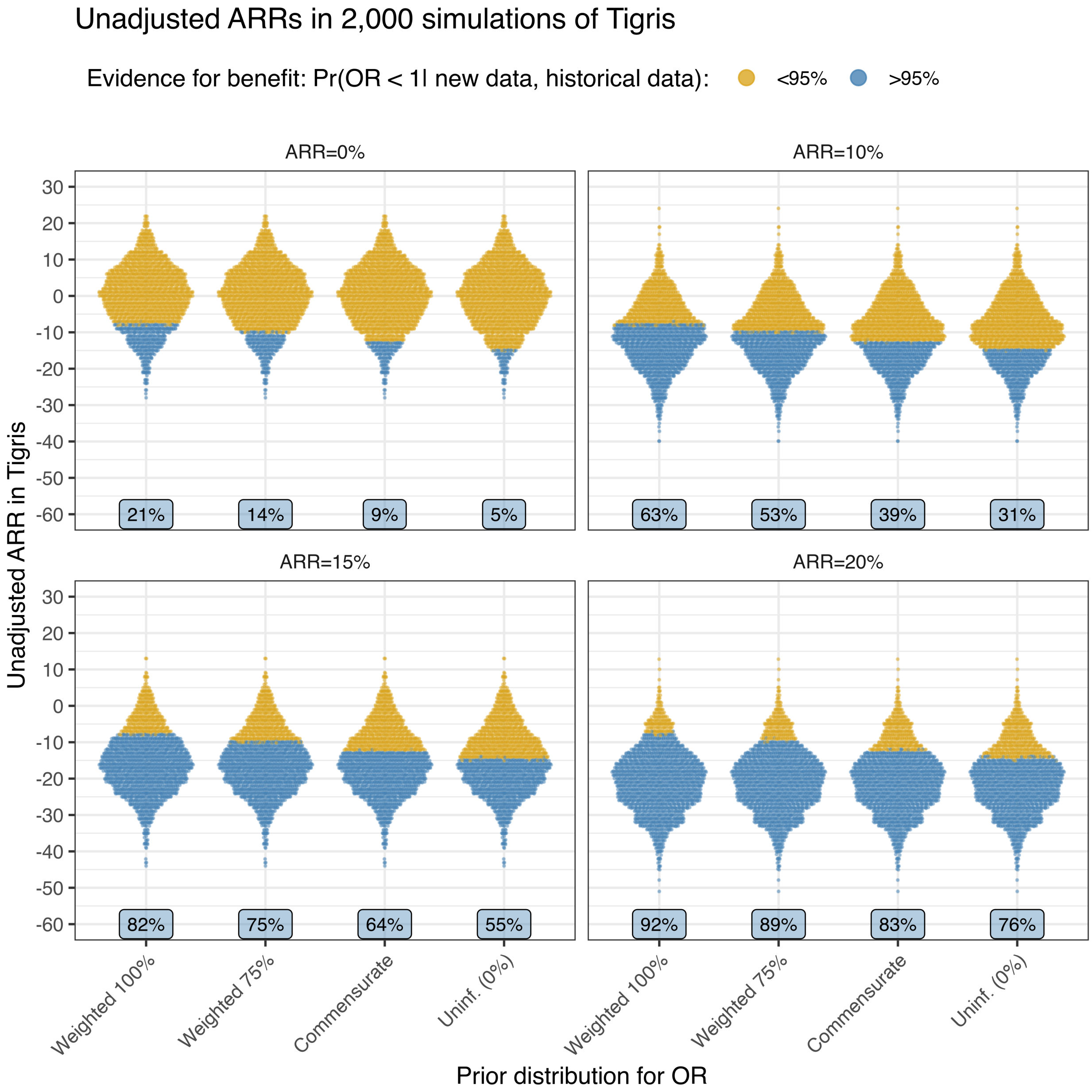

Figure S3: Unadjusted odds ratios in 2,000 simulated trials with a baseline risk of 50%. Blue labels indicate the percentages of simulated trials where we conclude benefit (i.e., the power) for the corresponding absolute risk reduction and use of historical data.

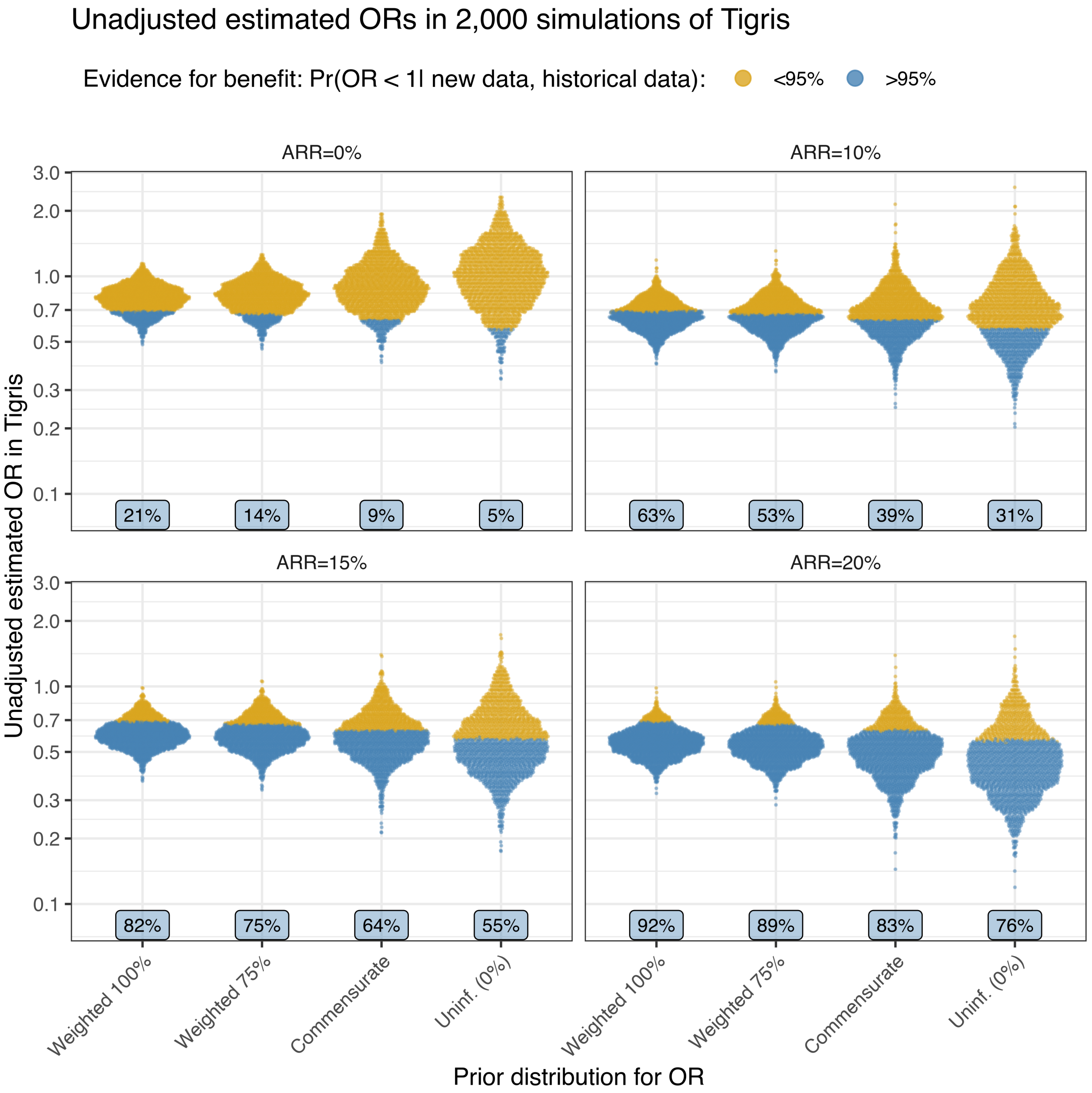

4. Tigris Trial Protocol Synopsis

A Prospective, Multicenter, Randomized, Open-Label Study to Evaluate the Efficacy and Safety of PMX Cartridge in Addition to Standard Medical Care for Patients with Endotoxemic Septic Shock:

TIGRIS TRIAL

Protocol Number: SDI-PMX-NA003

Protocol Version: 4.2

Version Date: 18 Nov-2021

www.spectraldx.com

Protocol Synopsis

**Study Title:**<br>A Prospective, Multicenter, Randomized, Open-Label Study to Evaluate the Efficacy and Safety of PMX Cartridge in Addition to Standard Medical Care for Patients with Endotoxemic Septic Shock: TIGRIS Trial
**Study Number and Acronym:**<br>SDI-PMX-NA003 TIGRIS
**Primary Objectives:**<br>The primary objective is to compare the safety and efficacy of the PMX cartridge (Toraymyxin) based on mortality at 28 days in patients with septic shock and endotoxemia who are treated with standard medical care plus the use of the PMX cartridge, versus patients who receive standard medical care alone.
**Secondary Objectives:**<br>The secondary objectives of the trial are as follows:<br>• To compare changes in mean arterial blood pressure (MAP) from Day 0 to Day 3 in each group<br>• To compare the changes in vasopressor doses from Day 0 to Day 3 in each group<br>• To compare the survival time from baseline to death within 28 days in each group<br>• To compare mortality at 28 days post baseline for patients with baseline norepinephrine dose >0.1 mcg/kg/min in each group<br>• To compare mortality at 14 days post baseline in each group<br>• To compare total duration of vasopressor use from Day 0 to Day 3 in each group
**Study Phase:**<br>Phase III
**Number of Centers:**<br>Approximately 15 in the USA
**Number of Subjects:**<br>Goal is to recruit 150 subjects
**Study Design:**<br>Prospective, multicenter, randomized, open-label study of standard of care plus the PMX cartridge versus standard of care alone.
**Investigational Device:**<br>The TORAYMYXIN PMX-20R (PMX cartridge) is a single-use extracorporeal hemadsorption device to remove endotoxin from patients' blood through direct hemadsorption.

**Treatment Intervention:**<br>Two (2) PMX cartridges will be administered approximately 24 hours apart to those subjects randomized to the treatment arm. Each treatment will target 2 hours with a minimum of 1 ½ hours, at a flow rate of approximately 100 mL/minute, (range of 80 to 120 mL/minute).<br>Subjects in both the treatment and control arms of the study will continue to receive standard medical care for septic shock.
**Patient Population:**<br>Intensive Care Unit subjects with septic shock and endotoxemia.

Inclusion Criteria:

Subjects who meet the following criteria (and have a signed informed consent) will be allowed into the study:

Age ≥18 years of age

Hypotension requiring vasopressor support: Requirement for at least one of the vasopressors listed below, at the dose shown below, for *at least* 2 continuous hours and no more than 30 hours*

a. Norepinephrine > 0.05mcg/kg/min
b. Dopamine > 10 mcg/kg/min
c. Phenylephrine > 0.4 mcg/kg/min
d. Epinephrine > 0.05 mcg/kg/min
e. Vasopressin > 0.03 units/min
f. Vasopressin (any dose) in combination with another vasopressor listed above

The subject must have received intravenous fluid resuscitation of a minimum of 30mL/kg administered within 24 hours of eligibility

Documented or suspected infection defined as definitive or empiric intravenous antibiotic administration

The subject must have a screening multi-organ dysfunction score (MODS) >9 **OR** a sequential organ failure assessment (SOFA) >11, in the event a complete MODS cannot be obtained due to missing measurements**

Endotoxin Activity Assay between ≥ 0.60 to <0.90 EA units

Evidence of at least 1 of the following criteria for new onset organ dysfunction that is considered to be due to the acute illness:

g. Requirement for positive pressure ventilation via an endotracheal tube or tracheostomy tube
h. Thrombocytopenia defined as acute onset of platelet count <150,000μ/L or a reduction of 50% from prior known levels
i. Acute oliguria defined as urine output <0.5mL/kg/hr for at least 6 hours despite adequate fluid resuscitation

* When determining the eligible dose of vasopressors for a subject whose measured body weight is >100 kg, the maximum weight of 100 kg (220 lbs) will be used. This maximum weight applies to both males and females.
** Subjects with MODS ≤ 9 who have a complete MODS will be excluded from the trial even if they have a SOFA >11.

Exclusion Criteria:

1. Inability to obtain an informed consent from the subject, family member or an authorized surrogate
2. Lack of commitment for full medical support
3. Inability to achieve or maintain a minimum mean arterial pressure (MAP) of ≥ 65mmHg despite vasopressor therapy and fluid resuscitation
4. Subject has end-stage renal disease and requires chronic dialysis
5. There is clinical support for non-septic shock such as:
   a. Acute pulmonary embolus
   b. Transfusion reaction
   c. Severe congestive heart failure (e.g. NYHA Class IV, ejection fraction < 35%)*
6. Subject has had chest compressions as part of CPR during this hospitalization without immediate return to communicative state
7. Subject has had an acute myocardial infarction (AMI) within the past 4 weeks
8. Subject has uncontrolled hemorrhage (acute blood loss requiring > 3 UPC in the past 24 hours)
9. Major trauma within 36 hours of screening
10. Subject has severe granulocytopenia (leukocyte count less than 500 cells/mm^3) or severe thrombocytopenia (platelet count less than 30,000 cells/mm^3)
11. HIV infection in association with a last known or suspected CD4 count of <50/mm^3
12. Subject's baseline state is non-communicative
13. Subject has sustained extensive third-degree burns within the past 7 days
14. Body weight < 35 kg (77 pounds)
15. Known hypersensitivity to Polymyxin B
16. Subject has known sensitivity or allergy to heparin or has a history of heparin associated thrombocytopenia (H.I.T.)
17. Subject is currently enrolled in an investigational drug or device trial
18. Subject has been previously enrolled in the current trial
19. Any other condition, that in the opinion of the investigator, would preclude the subject from being a suitable candidate for enrollment, such as end-stage chronic illness (eg. lack of source control and bowel necrosis) with no reasonable expectation of survival to hospital discharge

* Please note that an ejection fraction of <35% does not automatically exclude the subject. This ejection fraction example is intended to describe chronic severe congestive heart failure NYHA Class IV only.

**Study Procedures:**<br><br>This is a prospective, multicenter, randomized, open-label trial of standard medical care plus the PMX cartridge versus standard medical care alone, in subjects with endotoxemia and septic shock. Subjects in critical care areas will be assessed for septic shock using known or suspected infection, multiple organ failure, fluid resuscitation and hypotension requiring vasopressor support as primary criteria. Subjects will meet all entry criteria for study if endotoxin activity is within the range of ≥ 0.60 to <0.90.<br><br>Eligible and consented subjects will be randomized to receive either the PMX cartridge (administered twice for 1½ to 2 hours per treatment session approximately 24 hours apart) plus standard medical care or standard medical care alone. For all subjects in whom treatment has been initiated, a follow-up visit (if they are still in the hospital) or a telephone call will be completed at Day 28 (or later) to determine their mortality status. In surviving subjects, a follow-up visit or telephone call to determine their mortality status will also take place at approximately three months (i.e. Day 90) and 12 months after the subject was randomized.
**Study Duration:**<br><br>The duration of treatment and active follow-up for each subject will be from the time of treatment until 12 months post-treatment. Study enrollment is expected to be complete in 2023.
**Number of Assessments:**<br><br>There are 9 assessments including Eligibility, Baseline (Day 0), Days 1, 2, 3 and 4, then Day 28, Day 90 and 12 month.
**Safety Assessments:**<br><br>• Incidence of adverse events (AE, SAE, UADE) from the initiation of treatment up to the end of Day 4<br>• Incidence of treatment related adverse events (defined as possibly, probably or definitely related to the PMX cartridge, venous access for the purpose of the study or heparin use for the purpose of the study) from initiation of treatment until study completion<br>• Changes in blood chemistry, hematology and coagulation parameters

4. References

1. Romaschin AD, Obiezu-Forster CV, Shoji H, Klein DJ. Novel Insights into the Direct Removal of Endotoxin by Polymyxin B Hemoperfusion. *Blood purification.* 2017;44(3):193-197.
2. Cuturi SL, De Rosa S, Ferrer R, Ruiz-Rodriguez JC, Forfori F, Ronco C. Endotoxin activity trend and multi-organ dysfunction in critically ill patients with septic shock, who received Polymyxin-B hemadsorption: A multicenter, prospective, observational study. *Artif Organs.* 2023;47:1361-1370.
3. Marshall JC, Foster D, Vincent JL, et al. Diagnostic and prognostic implications of endotoxemia in critical illness: results of the MEDIC study. *J Infect Dis.* 2004;190(3):527-534.
4. Fujimori K, Tarasawa K, Fushimi K. Effectiveness of polymyxin B hemoperfusion for sepsis depends on the baseline SOFA score: a nationwide observational study. *Ann Intensive Care.* 2021;11(1):141.
5. Dellinger RP, Bagshaw SM, Antonelli M, et al. Effect of Targeted Polymyxin B Hemoperfusion on 28-Day Mortality in Patients With Septic Shock and Elevated Endotoxin Level: The EUPHRATES Randomized Clinical Trial. *JAMA.* 2018;320(14):1455-1463.
6. Jiang H, Kulkarni PM, Mallinckrodt CH, Shurzinske L, Molenberghs G, Lipkovich I. Covariate Adjustment for Logistic Regression Analysis of Binary Clinical Trial Data. *Stat Biopharm Res.* 2017;9:126-134.
7. Hernandez AV, Steyerberg EW, Habbema JD. Covariate adjustment in randomized controlled trials with dichotomous outcomes increases statistical power and reduces sample size requirements. *J Clin Epidemiol.* 2004;57(5):454-460.
8. Negassa A, Hanley JA. The effect of omitted covariates on confidence interval and study power in binary outcome analysis: a simulation study. *Contemp Clin Trials.* 2007;28(3):242-248.
